# Cepheid Xpert^®^ Flu/RSV and Seegene Allplex^™^ RP1 show high diagnostic agreement for the detection of influenza A/B and respiratory syncytial viruses in clinical practice

**DOI:** 10.1111/irv.12799

**Published:** 2020-08-20

**Authors:** Nasir Wabe, Robert Lindeman, Jeffrey J. Post, William Rawlinson, Melissa Miao, Johanna I. Westbrook, Andrew Georgiou

**Affiliations:** ^1^ Centre for Health Systems and Safety Research Australian Institute of Health Innovation Macquarie University North Ryde NSW Australia; ^2^ NSW Health Pathology Chatswood NSW Australia; ^3^ Department of Infectious Diseases Prince of Wales Hospital and Community Health Services Randwick NSW Australia; ^4^ Prince of Wales Clinical School University of New South Wales Kensington NSW Australia; ^5^ NSW Health Pathology Randwick Prince of Wales Hospital and Community Health Services Randwick NSW Australia; ^6^ School of Medical Sciences School of Biotechnology and Biomolecular Sciences University of New South Wales Kensington NSW Australia

**Keywords:** Allplex^™^ RP1, diagnostic agreement, molecular assays, Xpert^®^ Flu/RSV XC

## Abstract

**Background:**

Molecular assays based on reverse transcription‐polymerase chain reaction (RT‐PCR) provide reliable results for the detection of respiratory pathogens, although diagnostic agreement varies. This study determined the agreement between the RT‐PCR assays (Xpert^®^ Flu/RSV vs Allplex^™^ RP1) in detecting influenza A, influenza B, and respiratory syncytial viruses (RSVs) in clinical practice.

**Methods:**

We retrospectively identified 914 patient encounters where testing with both Xpert^®^ Flu/RSV and Allplex^™^ RP1 was undertaken between October 2015 and September 2019 in seven hospitals across New South Wales, Australia. The diagnostic agreement of the two assays was evaluated using positive percent agreement, negative percent agreement, and prevalence and bias‐adjusted kappa.

**Results:**

The positive percent agreement was 95.1% for influenza A, 87.5% for influenza B, and 77.8% for RSV. The negative percent agreement was 99.4% for influenza A, 99.9% for influenza B, and 100% for RSV. The prevalence and bias‐adjusted kappa was 0.98 for influenza A, 0.99 for influenza B, and 0.97 for RSV. In a sensitivity analysis, the positive percent agreement values were significantly higher during the non‐influenza season than the influenza season for influenza B and RSV.

**Conclusions:**

The Xpert^®^ Flu/RSV and Allplex^™^ RP1 demonstrated a high diagnostic agreement for all three viruses assessed. The seasonal variation in the positive percent agreement of the two assays for influenza B and RSV may have been due to lower numbers assessed, variability in the virology of infections outside the peak season, or changes in the physiology of the infected host in different seasons.

## 1 INTRODUCTION

Acute respiratory infections due to influenza and respiratory syncytial viruses (RSVs) have a significant health and economic burden in Australia^1^, ^2^ and internationally.^3^, ^4^ Worldwide, influenza is implicated in approximately two percent of all respiratory deaths,^5^ with an official World Health Organization estimate of 290 000‐650 000 seasonal influenza‐associated deaths globally each year.^6^ In 2017, the influenza virus was implicated in an estimated 11.5% of episodes of care for lower respiratory tract infection, including 9.5 million hospitalizations and 81.5 million hospital days worldwide.^4^ RSV is a leading cause of acute lower respiratory tract infections in infants and children throughout the developed and developing world.^3^ In 2015, RSV was implicated in 33.1 million healthcare episodes, 3.2 million hospital admissions, and 59 600 in‐hospital deaths in children younger than 5 years, as well as 1.4 million hospital admissions and 27 300 in‐hospital deaths in children younger than 6 months.^3^

Molecular assays based on reverse transcription‐polymerase chain reaction (RT‐PCR) are considered to be the best and fastest available assays to detect respiratory viruses. In Australia, Seegene's Allplex^™^ Respiratory Panel (RP; South Korea) and Cepheid's Xpert^®^ Flu/RSV (USA) are two of the available RT‐PCR tests for the detection of influenza A, influenza B and RSV. Allplex^™^ RP is a multiplex real‐time one‐step RT‐PCR assay that detects up to 16 respiratory viruses and provides real‐time influenza A subtyping.^7^ It comprises three viral panels and one bacterial panel. The first of these viral panels (the Allplex^™^ RP1) detects influenza A and subtypes (H1, H1pdm09, and H3), influenza B, and RSV (types A and B). Although the Allplex^™^ RP offers comprehensive testing for respiratory viruses, it is undertaken by a central laboratory and specimens are tested in batches (processing multiple specimens simultaneously) rather than through continuous needs‐based testing. Therefore, it has a test turnaround time of 1‐4 days depending upon the frequency of batch testing and location of the hospital.^8^

The Xpert Flu/RSV XC is an automated, multiplex real‐time, RT‐PCR assay for the detection of influenza A, influenza B, and RSV.^9^ It has a faster turnaround time of 1‐4 hours,^8^, ^10^ but unlike the Allplex^™^ RP1, Xpert^®^ Flu/RSV cannot discriminate among influenza A virus subtypes. Nevertheless, the rapid identification of respiratory viruses has the potential to improve patient outcomes by supporting clinical decision‐making around antimicrobial use.^11^ More rapid identification could also improve clinical processes, including optimizing bed management and infection control^12^ as well as minimizing unnecessary ancillary test utilizations,^8^, ^10^, ^13^ with subsequent reductions to healthcare costs.^14^

All molecular assays based on RT‐PCR provide reliable results for the detection of respiratory viruses but are not always in diagnostic agreement. Previous studies reported the performance of Xpert^®^ Flu/RSV and Allplex^™^ RP1 against other reference assays.^7^, ^9^, ^15^, ^16^, ^17^, ^18^, ^19^, ^20^ However, to the best of our knowledge, no prior published studies have compared the Xpert^®^ Flu/RSV and Allplex^™^ RP1 in clinical practice. The objective of the study was to determine the diagnostic agreement between Xpert^®^ Flu/RSV vs Allplex^™^ RP1 for the detection of influenza A, influenza B, and RSV in the hospital emergency department (ED) or inpatient settings.

## 2 MATERIAL AND METHODS

### 2.1 Study design and setting

We conducted a retrospective observational study utilizing 4 years of data from October 2015 to September 2019 extracted from seven public hospitals (Hospitals A‐G) in New South Wales, Australia (six general hospitals and one children's hospital [Hospital D]). Xpert^®^ Flu/RSV was introduced to four of the study hospitals (Hospitals A‐D) in July 2017, while Hospitals E‐G have used the test since October 2015. Therefore, the data before July 2017 were only from the other three hospitals (Hospitals E‐G).

### 2.2 Participants and data sources

**FIGURE 1:**
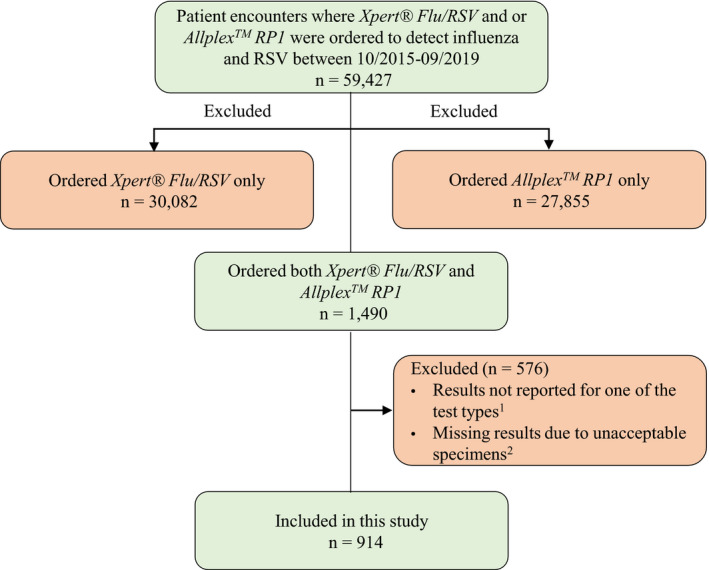
Patient selection flowchart. ^1^Reasons for not reporting the results were not recorded; ^2^Unacceptable specimen (eg, unlabeled, unsuitable or mislabeled) in either Xpert^®^ Flu/RSV or Allplex^™^ RP1 sample

Eligibility criteria included patients for whom both Xpert^®^ Flu/RSV and Allplex^™^ RP1 tests were ordered at the same time for the same episode of care. Exclusion criteria specified patients for whom: (a) only one of the assays was ordered, (b) both assays were ordered but at different times (eg, Xpert^®^ Flu/RSV while the patient was in the ED and Allplex^™^ RP1 while the patient was in the inpatient ward), and (c) both assays were ordered but the result of one of the assays was not reported or missing due to unacceptable specimens (Figure 1).

The data used for this study were sourced from the Laboratory Information System (LIS) of each hospital. The LIS contains laboratory test order information including, but not limited to, patient age and sex, patient Medical Reference Number, test order episode identification, type of tests ordered, location of the order, test results and specimen types, as well as the date and time a specimen is collected, received at the laboratory and a verified result available. All study hospitals used one LIS and thus had similar test catalogs and configurations.

### 2.3 Test methods

Detailed information regarding the use of the two assays has been reported in our previous studies.^8^, ^10^, ^21^ Briefly, Allplex^™^ RP has been used as a referral test at a large central laboratory located at Hospital B and all other hospitals sent samples to this laboratory for analysis. Testing was performed in batches once or twice depending upon demand.^8^ As Allplex^™^ RP was offered as three viral panels, in addition to panel 1, clinicians had the option of ordering panel 2 (which detects adenovirus, metapneumovirus, enterovirus, and parainfluenza viruses) and panel 3 (which detects bocaviruses, coronaviruses, and rhinoviruses) depending on the clinical scenario. Unlike AllplexTM RP, testing using the Xpert^®^ Flu/RSV was performed at local hospital laboratories avoiding the need for sending the specimen to the central laboratory. While both assays were available to clinicians during the study period, the use of Xpert^®^ Flu/RSV was recommended to be used by the laboratory service for patients at high risk of influenza, where a result was required more urgently. This included intensive care patients with influenza‐like illness, immunocompromised patients with influenza‐like illness, ED patients with a significant respiratory infection and isolation requirement. Nasopharyngeal swabs and nasopharyngeal aspirates were the two main types of specimens that have been used for both Xpert^®^ Flu/RSV and AllplexTM RP1. Sputum or bronchial lavages were also used in very few cases.

### 2.4 Statistical analysis

A two‐by‐two contingency table comparing the results of Xpert^®^ Flu/RSV against Allplex^™^ RP1 was created. The diagnostic agreement of the two assays was evaluated using positive percent agreement (PPA), negative percent agreement (NPA), kappa, and prevalence and bias‐adjusted kappa (PABAK) along with their 95% confidence intervals (CI). PPA (which is analogous to sensitivity) was calculated by dividing the *number of Allplex^™^ RP1^+^ and Xpert^®^+* cases by *total Allplex^™^ RP1^+^* cases. NPA (which was analogous to specificity) was calculated by dividing the *number of Allplex^™^ RP1− and Xpert^®^−* cases by *total Allplex^™^ RP1−* cases. The values of kappa can range from −1 to 1 and can be interpreted as <0.2 as none to slight; 0.21‐0.4 as fair; 0.41‐0.6 as moderate; 0.61‐0.8 as substantial; and 0.81‐1.0 as almost perfect agreement.^22^ Because kappa can be affected by disease prevalence and potential bias between the assays, PABAK was reported to account for these influences.^23^ This is particularly important given the low prevalence of respiratory viruses in our sample. Bias in the context of this study is said to occur when the two assays differ in the frequency of the detection of a given virus in the study sample.^23^

Baseline factors associated with discordant results between the two assays were determined using *penalized logistic regression* as it reduces the small‐sample bias and is thus suitable for modeling low prevalence binary outcomes.^24^ A discordant result (yes/no) was defined as discrepancies between the results of the two assays in at least one of the three viruses (ie, Allplex^™^ RP1^+^ and Xpert^®^− and vice versa).

### 2.5 Ethical approval

Ethical approval for the study was granted by the Human Research Ethics Committee of the South Eastern Sydney Local Health District (reference, HREC/16/POWH/412).

## 3 RESULTS

### 3.1 Participants

A total of 914 patient encounters fulfilled the inclusion criteria (Figure 1). The median age was 28 years, and 55.2% (n = 505) were male. A nasopharyngeal swab was the most common specimen type (Table 1).

**TABLE 1:** Baseline characteristics (n = 914)

Variable	N (%)
Gender	
Female	409 (44.8)
Male	505 (55.2)
Age, median (IQR)	28 (2‐71)
Age category	
<18 y	429 (46.9)
≥18 y	485 (53.1)
Influenza season (June‐September)	
No	502 (54.9)
Yes	412 (45.1)
Setting where tests were ordered	
Emergency departments	516 (56.5)
Inpatient wards (including ICUs)	398 (43.5)
Specimen type	
NP swab (both assays)	460 (50.3)
NP aspirate (both assays)	307 (33.6)
NP swab (Allplex^™^ RP1) and NP aspirate (Xpert^®^ Flu/RSV)	109 (11.9)
NP swab (Xpert^®^ Flu/RSV) and NP aspirate (Allplex^™^ RP1)	35 (3.9)
Sputum or bronchial lavages	3 (0.3)
Year (Oct 2015‐Sept 2019)	
2015	61 (6.7)
2016	304 (33.3)
2017	157 (17.2)
2018	227 (24.8)
2019	165 (18.1)
Hospital	
A	254 (27.8)
B	66 (7.2)
C	109 (11.9)
D	47 (5.1)
E	282 (30.9)
F	27 (3.0)
G	129 (14.1)

Abbreviations: ICU, intensive care unit; IQR, interquartile range; NP, nasopharyngeal.

### 3.2 Test results

**FIGURE 2:**
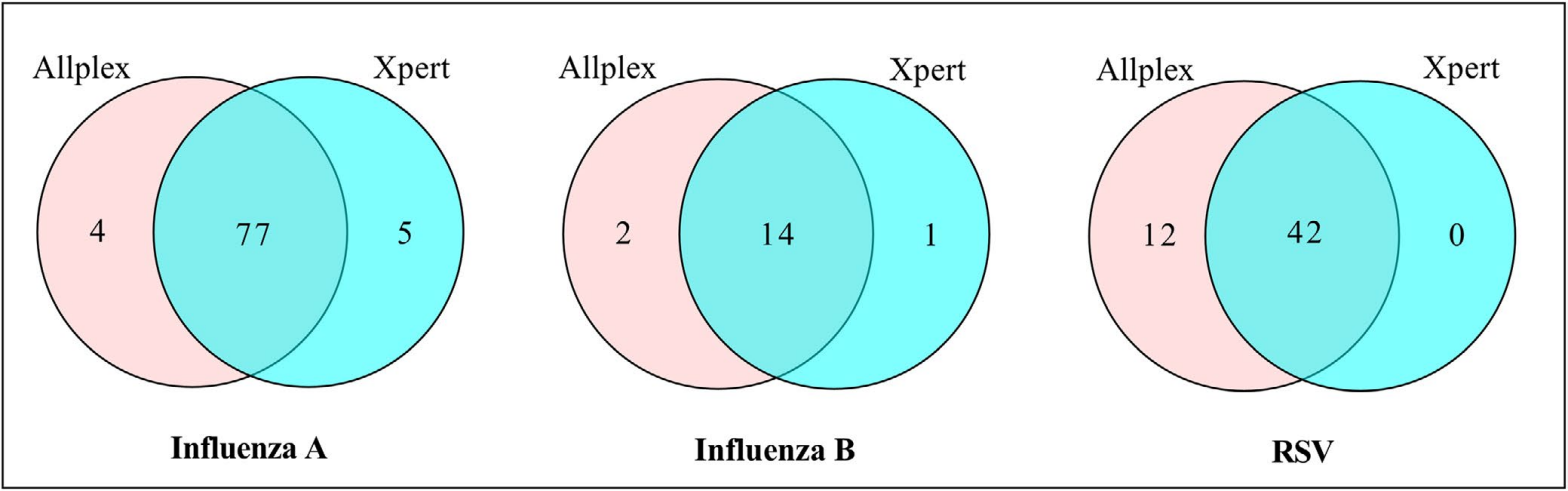
Venn diagram showing the number of influenza A, influenza B, and RSV detected by Xpert^®^ Flu/RSV and Allplex^™^ RP1

The median turnaround time from specimen receipt to authorized result was 3.3 hours (ranged from 1.5 to 10.4 hours across hospitals) for the Xpert^®^ Flu/RSV and 39.6 hours (ranged from 23.4 to 69.4 hours across hospitals) for the Allplex^™^ RP1. The distribution of positive specimens for each assay is shown in Figure 2. Both Xpert^®^ Flu/RSV and Allplex^™^ RP1 detected roughly similar proportions of cases of influenza A or B but Allplex^™^ RP1 detected slightly more RSV compared to Xpert^®^ Flu/RSV. Of the total 914 specimens, 82 (9.0%) were positive for influenza A with Xpert and 81 (8.9%) were positive with Allplex; 15 (1.6%) were positive for influenza B with Xpert and 16 (1.8%) were positive with Allplex and 42 (4.6%) were positive for RSV with Xpert and 54 (5.9%) were positive with Allplex.

### 3.3 Diagnostic agreement

The diagnostic agreement measures are shown in Table 2. The PPA was 95.1% for influenza A, 87.5% for influenza B, and 77.8% for RSV. The NPA values were very high (>99%) for all viruses. The PABAK values were also high (≥0.97) for all viruses indicating a high level of agreement between the two assays (Table 2).

**TABLE 2:** Diagnostic agreement of Xpert^®^ Flu/RSV with Allplex^™^ RP1 (n = 914)

	Influenza A			Influenza B			RSV		
	Allplex^™^ RP1			Allplex^™^ RP1			Allplex^™^ RP1		
Xpert^®^ Flu/RSV	+	−	Total	+	−	Total	+	−	Total
+	77	5	82	14	1	15	42	0	42
−	4	828	832	2	897	899	12	860	872
Total	81	833	914	16	898	914	54	860	914
PPA (95% CI)	95.1 (87.8‐98.6)			87.5 (61.7‐98.4)			77.8 (64.4‐88.0)		
NPA (95% CI)	99.4 (98.6‐99.8)			99.9 (99.4‐100.0)			100 (99.6‐100.0)		
Kappa (95% CI)	0.94 (0.90‐0.98)			0.90 (0.79‐1.00)			0.87 (0.79‐0.94)		
PABAK (95% CI)	0.98 (0.97‐0.99)			0.99 (0.98‐1.00)			0.97 (0.96‐0.99)		

Abbreviations: NPA, negative percent agreement, PABAK, prevalence and bias‐adjusted kappa; PPA, positive percent agreement; RSV, respiratory syncytial virus.

### 3.4 Factors associated with diagnostic disagreement

Overall, 23 specimens showed a discordant result. One of these specimens showed discrepancies in results for more than one virus (Table S1). Gender, age category, setting where tests were ordered or the type of specimen was not associated with a discordant result. In other words, there were no significant differences in the results of the two tests across these characteristics. However, influenza season status was significantly associated with a discordant result. The difference between the two assays was higher during influenza season compared to the non‐influenza season. Xpert^®^ Flu/RSV and Allplex^™^ RP1 were 3.38 times more likely to have discordant results during the influenza season than the non‐influenza season (Table 3).

**TABLE 3:** Factors associated with disagreement between Xpert^®^ Flu/RSV and Allplex^™^ RP1 in the detection of influenza A, influenza B, or RSV (univariate analysis)

	Concordant (+/+ or −/−)	Discordant (∓ or ±)	Total	OR (95% CI)	*P*‐value
Gender					
Female (Ref)	401	8	409	1.49 (0.64‐3.48)	.354
Male	490	15	505		
Total	891	23	914		
Age category					
<18 y (Ref)	418	11	429	0.96 (0.43‐2.16)	.923
≥18 y	473	12	485		
Total	891	23	914		
Influenza season					
No (Ref)	496	6	502	3.38 (1.36‐8.40)	.009
Yes	395	17	412		
Total	891	23	914		
Setting					
Inpatient (Ref)	392	6	398	2.11 (0.85 = 5.26)	.107
ED	499	17	516		
Total	891	23	914		
Specimen type					
NP swab (Ref)	447	13	460		
NP aspirate	301	6	307	0.71 (0.23‐1.84)	.487
Other^a^	143	4	147	1.04 (0.35‐3.07)	.944
Total	891	23	914		
Hospital					
A (Ref)	247	7	254		
B	66	0	66	0.25 (0.01‐4.40)	.342
C	109	0	109	0.15 (0.01‐2.66)	.196
D	43	4	47	3.41 (1.02‐11.47)	.047
E	275	7	282	0.99 (0.32‐2.51)	.838
F	24	3	27	4.71 (1.24‐17.91)	.023
G	127	2	129	0.64 (0.15‐2.75)	.555

Abbreviations: ED, emergency department; NP, nasopharyngeal.

^a^ NP swab (Allplex) and NP aspirate (Xpert) or NP aspirate (Allplex) and NP swab (Xpert) or the use of sputum or bronchial lavages in one of the assays.

### 3.5 Sensitivity analysis

Sensitivity analysis by influenza season status was conducted as it was associated with the discrepancy between the results of the tests. The main discrepancy was with respect to influenza B and RSV. The PPA values were higher during the non‐influenza season for influenza B and RSV. For example, the PPA values for RSV were 50% and 96.9% during influenza and non‐influenza seasons, respectively. For influenza A, the measures are roughly the same as each other (Table 4).

**TABLE 4:** Diagnostic agreement of Xpert^®^ Flu/RSV with Allplex^™^ RP1 by influenza season

	Influenza A						Influenza B						RSV					
	Influenza season (n = 412)			Non‐influenza season (n = 502)			Influenza season (n = 412)			Non‐influenza season (n = 502)			Influenza season (n = 412)			Non‐influenza season (n = 502)		
	Allplex^™^ RP1			Allplex^™^ RP1			Allplex^™^ RP1			Allplex^™^ RP1			Allplex^™^ RP1			Allplex^™^ RP1		
Xpert^®^ Flu/RSV	+	−	Total	+	−	Total	+	−	Total	+	−	Total	+	−	Total	+	−	Total
+	40	2	42	37	3	40	9	1	10	5	0	5	11	0	11	31	0	31
−	2	368	370	2	460	462	2	400	402	0	497	497	11	390	401	1	470	471
Total	42	370	412	39	463	502	11	401	412	5	497	502	22	390	412	32	470	502
PPA (95% CI)	95.2 (83.8‐99.4)			94.9 (82.7‐99.4)			81.8 (48.2‐97.7)			100.0 (47.8‐100.0)			50.0 (28.2‐71.8)			96.9 (83.8‐99.9)		
NPA (95% CI)	99.5 (98.1‐99.9)			99.4 (98.1‐99.9)			99.8 (98.6‐100.0)			100.0 (99.3‐100.0)			100.0 (99.1‐100.0)			100.0 (99.2‐100.0)		
Kappa (95% CI)	0.95 (0.90‐0.99)			0.93 (0.87‐0.99)			0.85 (0.69‐1.00)			1.00 (1.00‐1.00)			0.65 (0.46‐0.84)			0.98 (0.95‐1.00)		
PABAK (95% CI)	0.98 (0.96‐0.99)			0.98 (0.96‐0.99)			0.98 (0.97‐1.00)			1.00 (1.00‐1.00)			0.95 (0.92‐0.98)			1.00 (0.99‐1.00)		

Abbreviations: NPA, negative percent agreement; PABAK, prevalence and bias‐adjusted kappa; PPA, positive percent agreement; RSV, respiratory syncytial virus.

## 4 DISCUSSION

### 4.1 Key findings

We retrospectively evaluated the agreement of two PCR‐based assays (Xpert^®^ Flu/RSV vs Allplex^™^ RP1) for the detection of influenza A, influenza B, and RSV. The key finding was that the Xpert^®^ Flu/RSV demonstrated a high level of agreement with Allplex^™^ RP1 for all viruses with ≥0.97 PABAK values. The NPA was generally very high for all viruses. The PPA was relatively high for influenza A (95.1%), followed by influenza B (87.5%) and RSV (77.8%). We found that the PPA value was substantially higher during the non‐influenza season than the influenza season, particularly for influenza B and RSV.

### 4.2 Interpretation and comparison with existing literature

No previous studies comparing the Xpert^®^ Flu/RSV and Allplex^™^ RP, against which to compare our findings, were identified. However, the performance of each assay has previously been evaluated against other molecular assays. Xpert^®^ Flu/RSV has been compared to quality control samples,^15^ single‐plex real‐time RT‐PCR,^19^ a laboratory‐developed assay,^18^ BioFire FilmArray (USA),^20^ and GenMark Diagnostics eSensor RVP (USA),^9^ and reported to have the following measures: (a) 91%‐100% PPA/sensitivity and 99%‐100% NPA/specificity for influenza A; (b) 80%‐100% PPA/sensitivity and 99%‐100% NPA/specificity for influenza B; and (iii) 91%‐100% PPA/sensitivity and 99%‐100% NPA/specificity for RSV.^9^, ^15^, ^18^, ^19^, ^20^ Allplex^™^ RP has been compared to AdvanSure (Korea),^7^ Anyplex II RV16 (Korea),^17^ Simplexa^™^ Flu A/B & RSV (USA),^16^ and quality control samples^15^ and reported to have 90%‐98% PPA/sensitivity and 100% NPA/specificity for influenza A, 89%‐100% PPA/sensitivity and 100% NPA/specificity for influenza B, and 95%‐100% PPA/sensitivity and 100% NPA/specificity for RSV.^7^, ^15^, ^16^, ^17^

A study by Gimferrer et al^15^ is the only study that has assessed both Xpert^®^ Flu/RSV and Allplex^™^ RP1 against the same reference assay. That study evaluated three molecular tests including Xpert^®^ Flu/RSV, Allplex^™^ RP1, and Prodesse ProFlu+/ProFAST+ (USA) against laboratory‐confirmed quality control samples (239 positives and 77 negatives). Although the study did not compare Xpert^®^ Flu/RSV and Allplex^™^ RP1 against each other, both tests demonstrated comparable sensitivity and specificity against quality control samples. Allplex^™^ RP1 showed a sensitivity of 90.2% for influenza A, 88.9% for influenza B, and 100% for RSV, while Xpert^®^ Flu/RSV showed a sensitivity of 91.2% for influenza A, 91.1% for influenza B, and 100% for RSV. Both tests demonstrated specificity values of 100% and slightly better sensitivity and specificity values than Prodesse ProFlu+/ProFAST+ for all three viruses.^15^

Positive percent agreement (which can be interpreted similarly as sensitivity) refers to the ability of a test to correctly diagnose all patients with a disease. It is an important measure to rule‐out a disease when the test result is negative.^25^ In general, our findings are consistent with the PPA/sensitivity values reported in previous studies for influenza A and B.^7^, ^9^, ^15^, ^16^, ^17^, ^18^, ^19^, ^20^ For RSV, however, although the NPA values are within the ranges of the previous reports,^7^, ^9^, ^15^, ^16^, ^17^, ^18^, ^19^, ^20^ Xpert^®^ Flu/RSV detected less RSV cases than Allplex^™^ RP1 (42 vs 54 positive cases) and the PPA was 77.8% in our study. This PPA value was lower than the findings of Gimferrer et al^15^ and other studies which reported PPA/sensitivity values of 91%‐100% for RSV.^9^, ^18^, ^19^, ^20^ Further studies may be needed to confirm the PPA of Xpert^®^ Flu/RSV for RSV using a prospective cohort design.

In Australia, although influenza viruses circulate year‐round, in most cases, influenza notifications peak between June and September. Influenza A was the predominant virus compared to influenza B throughout the 2016‐2018 influenza seasons in New South Wales.^26^, ^27^, ^28^ The type of circulating strains varied from season to season. For instance, in 2016 and 2017,^26^, ^27^ H3N2 was the most common influenza A strain, while in 2018,^28^ H1N1 was the predominant influenza A strain. For influenza B, both B/Yamagata lineage and B/Victoria lineage strains were circulating throughout the 2016‐2018 influenza seasons with B/Yamagata lineage strains more prevalent than B/Victoria lineage.^26^, ^27^, ^28^

In a sensitivity analysis by influenza season status in this study, the PPA values of the two assays were significantly higher during the non‐influenza season than the influenza season, particularly for influenza B and RSV. This seasonal variation may have been due to lower numbers assessed, variability in the virology of infections outside the peak season, or changes in infected host physiology in different seasons. No differences in PPA values were associated with any of the other variables.

### 4.3 Implications for practice

The main clinical implication of our finding is that there is no need to use both assays at the same time in clinical practice, given the high level of agreement between the two assays (with ≥0.97 PABAK values). Depending on the clinical scenario, clinicians can confidently choose one of the assays. Xpert^®^ Flu/RSV can detect three of the most important respiratory viruses (influenza A, influenza B, and RSV) although it cannot discriminate the subtypes of influenza A. As Xpert^®^ Flu/RSV offers a more rapid turnaround time than Allplex^™^ RP1, our findings suggest potential clinical and process advantages of the use of Xpert^®^ Flu/RSV, especially when influenza is the main suspected infection and an urgent result is needed. Allplex^™^ RP1 can be used when an urgent test result is not needed, if influenza subtyping is required or if there is a need to use Allplex^™^ RP1 along with other Seegene's viral panels to screen for multiple respiratory viruses.

### 4.4 Strengths and limitations

To the best of our knowledge, this is the first study to compare the Xpert^®^ Flu/RSV and Allplex^™^ RP1. The inclusion of diverse study populations from multiple sites including six general and one children's hospitals can be considered as the main strength of this study. The key limitation of this study was that, in the case of discrepant results between the two assays, a three‐way comparison using another confirmatory method has not been conducted to verify the results. This study utilized retrospective data from hospitals where Xpert^®^ Flu/RSV and Allplex^™^ RP1 were the main laboratory tests for the diagnosis of influenza and RSV. It was not possible to determine which of the two assays was correct in the case of discrepancies between the assays. The Allplex^™^ RP1 assay flexibility in assessing other non‐respiratory viruses at a lower unit cost, also means assessment of other causes of a patient's symptoms are potentially assessed. According to the local guideline for the use of Xpert^®^ Flu/RSV, its use was mainly reserved for patients at high risk of influenza where an urgent result was needed. The study population selected for this study may, therefore, differ in disease severity from other patients presenting to the hospitals with influenza‐like illnesses but were not eligible to receive the test, potentially introducing bias into the study. As this study was not designed to assess test cost or health economic outcomes, no conclusions around this can be drawn.

## 5 CONCLUSION

In conclusion, Xpert^®^ Flu/RSV XC and Allplex^™^ RP1 demonstrated a high diagnostic agreement for all three viruses assessed. The seasonal variation in the PPA of the two assays for influenza B and RSV may have been due to lower numbers assessed, variability in the virology of infections outside the peak season, or changes in infected host physiology in different seasons.

## CONFLICT OF INTERESTS

The authors declare that they have no conflict of interest.

## AUTHOR CONTRIBUTION

**Nasir Wabe:** Conceptualization (lead); Data curation (lead); Formal analysis (lead); Methodology (lead); Project administration (lead); Software (lead); Writing‐original draft (lead); Writing‐review & editing (equal). **Robert Lindeman:** Conceptualization (equal); Funding acquisition (equal); Resources (equal); Supervision (equal); Writing‐review & editing (equal). **Jeffrey J Post:** Conceptualization (equal); Investigation (equal); Resources (equal); Supervision (equal); Writing‐review & editing (equal). **William Rawlinson:** Conceptualization (equal); Investigation (equal); Resources (equal); Supervision (equal); Writing‐review & editing (equal). **Melissa Miao:** Conceptualization (equal); Project administration (equal); Writing‐original draft (supporting); Writing‐review & editing (supporting). **Johanna I Westbrook:** Funding acquisition (equal); Resources (equal); Supervision (equal); Writing‐review & editing (equal). **Andrew Georgiou:** Conceptualization (lead); Funding acquisition (lead); Resources (equal); Supervision (lead); Writing‐review & editing (equal).

## Supporting information

Table S1
