# Supplementary material for: Cepheid Xpert® Flu/RSV and Seegene Allplex™ RP1 show high diagnostic agreement for the detection of influenza A/B and respiratory syncytial viruses in clinical practice
Source: Influenza Other Respir Viruses. 2020 Aug 20;15(2):245–53. doi: 10.1111/irv.12799 (PMC7461471; doi:10.1111/irv.12799)
Supplement: Supplementary file 1 — Table S1 [file IRV-15-245-s001.docx]

**Appendix**

**Supplementary Table 1**: Characteristics of discrepant results (n=23). The subtypes of the virus detected (for Influenza A and RSV) where Allplex was used is indicated in the bracket.

| S.no | Influenza A | | Influenza B | | RSV | | Other Allplex RP ordered | Allplex panel 2/3 results if ordered |
| --- | --- | --- | --- | --- | --- | --- | --- | --- |
|  | Xpert | Allplex | Xpert | Allplex | Xpert | Allplex |  |  |
| 1 | Neg. | Neg. | Neg. | Neg. | Neg. | Pos. (A) | 2 and 3 | Rhinovirus |
| 2 | Neg. | Neg. | Neg. | Neg. | Neg. | Pos. (A) | 2 and 3 | Rhinovirus |
| 3 | Neg. | Pos. (H109) | Neg. | Neg. | Neg. | Neg. | 2 and 3 | All Neg. |
| 4 | Neg. | Neg. | Neg. | Neg. | Neg. | Pos. (A) | 2 and 3 | Rhinovirus |
| 5 | Neg. | Neg. | Neg. | Neg. | Neg. | Pos. (A) | 2 and 3 | Adenovirus and Rhinovirus |
| 6 | Pos. | Neg. | Neg. | Neg. | Neg. | Neg. | - |  |
| 7 | Pos. | Neg. | Neg. | Neg. | Neg. | Neg. | 2 and 3 | All Neg. |
| 8 | Neg. | Pos. (H109) | Neg. | Neg. | Neg. | Neg. | 2 and 3 | All Neg. |
| 9 | Neg. | Neg. | Pos. | Neg. | Neg. | Neg. | - |  |
| 10 | Neg. | Neg. | Neg. | Pos. | Neg. | Neg. | 2 and 3 | All Neg. |
| 11 | Pos. | Neg. | Neg. | Neg. | Neg. | Neg. | - |  |
| 12 | Neg. | Pos. (H09) | Neg. | Neg. | Neg. | Neg. | 2 and 3 | Parainfluenza virus |
| 13 | Neg. | Neg. | Neg. | Neg. | Neg. | Pos. (A) | 2 and 3 | Rhinovirus |
| 14 | Neg. | Neg. | Neg. | Neg. | Neg. | Pos. (A) | 2 and 3 | All Neg. |
| 15 | Pos. | Neg. | Neg. | Neg. | Neg. | Neg. | - |  |
| 16 | Neg. | Neg. | Neg. | Neg. | Neg. | Pos. (B) | 2 and 3 | Metapneumovirus,  Rhinovirus and Coronavirus |
| 17 | Neg. | Pos. (H3) | Neg. | Neg. | Neg. | Pos. (B) | 2 and 3 | Rhinovirus |
| 18 | Neg. | Neg. | Neg. | Neg. | Neg. | Pos. (B) | 2 and 3 | Adenovirus |
| 19 | Pos. | Neg. | Neg. | Neg. | Neg. | Neg. | - |  |
| 20 | Neg. | Neg. | Neg. | Neg. | Neg. | Pos. (B) | 2 and 3 | All Neg. |
| 21 | Neg. | Neg. | Neg. | Neg. | Neg. | Pos. (B) | 2 and 3 | All Neg. |
| 22 | Neg. | Neg. | Neg. | Neg. | Neg. | Pos. (B) | 2 and 3 | Corona virus |
| 23 | Neg. | Neg. | Neg. | Pos. | Neg. | Neg. | 2 and 3 | All Neg. |
